# Ocular manifestations for misdiagnosing acute angle closure secondary to lens subluxation

**DOI:** 10.3389/fmed.2024.1410689

**Published:** 2024-10-07

**Authors:** Xinyu Wang, Qian Wang, Wenqi Song, Yu Yang, Ruixuan Zhang, Gao Lifen, Hui Lu

**Affiliations:** Zibo Central Hospital, Zibo, Shandong, China

**Keywords:** acute primary angle closure, lens subluxation, anterior chamber depth difference, IOLMaster, glaucoma

## Abstract

**Introduction:**

The objective of this study was to assess the clinical characteristics and biometric parameters, as measured by the IOLMaster, of patients suffering from acute secondary angle closure due to zonular dialysis (ASAC-ZD) who were misdiagnosed with acute primary angle closure (APAC).

**Methods:**

In this retrospective study, 34 ASAC-ZD and 39 APAC eyes were examined. Sex, age, best-corrected visual acuity, axial length (AL), anterior chamber depth (ACD), anterior chamber depth standard deviation (ACDSD), lens thickness (LT), and lens thickness standard deviation (LTSD) were measured using the IOLMaster and compared between the two groups. In addition, the difference in ACD (ACD difference) between the affected eye and the contralateral eye was analyzed. Logistic regression analysis was performed to determine the predictive factors of lens subluxation. To determine the appropriate cutoff values for biometric parameters, ROC curves were constructed to distinguish between ASAC-ZD, APAC, and cataracts.

**Results:**

Compared to the APAC group, the ASAC-ZD group was younger (69.92 ± 9.345, 63.74 ± 6.947), had longer AL (22.39 ± 0.7852, 23.23 ± 1.168), shallower ACD (2.120 ± 0.2986, 1.889 ± 0.5167), higher ACDSD (7.605 ± 5.425, 9.941 ± 6.120), higher LTSD (28.00 ± 19.52, 39.79 ± 22.74), and larger ACD differences (−0.1249 ± 0.2349, −0.7306 ± 0.5332) in the affected eye. Younger age, longer AL, lower ACD, higher LTSD, and higher ACD differences were associated with lens subluxation in the univariate logistic regression analysis. ACD difference (*p* = 0.0003), age (*p* = 0.0024), and ACD (*p* = 0.0491) were significantly associated with lens subluxation in the multivariable logistic regression analysis. Furthermore, the ROC curve analysis showed that the cutoff values for lens subluxation were a difference in ACD of 0.225 mm and 1.930 mm.

**Conclusion:**

Asymmetric ACD in both eyes with normal AL and increasing ACDSD and LTSD may support the clinical diagnosis of lens subluxation.

## 1 Introduction

Acute angle closure is an ophthalmic emergency that, if left untreated, can lead to irreversible loss of vision, corneal endothelial decompensation, and secondary optic neuropathy (1, 2). Angle closure can be caused by several mechanisms, such as pupillary block and abnormal lens position (3, 4). Abnormal lens position, also known as lens subluxation, is often caused by rupture of the zonule, leading to secondary angle closure. Unexplained or traumatic lens subluxation may lead to the forward movement of the displaced lens, resulting in a shallow anterior chamber, secondary pupillary block, and occlusion of the anterior angle of the eye (5, 6). Symptoms and signs of ASAC with lens subluxation include nausea, vomiting, severe ocular pain, elevated intraocular pressure (IOP), corneal edema, and visual impairment, which are common in APAC patients.

Given a broad range of zonular ruptures or a specific history of eye trauma, the diagnosis can be standardized, as silt-lamp microscopy can easily detect typical signs of lens subluxation, including iridodonesis, a dislocated lens visible at the equator, and vitreous incarceration in the anterior chamber angle and pupil (7–9). However, due to a small range of zonular ruptures, the stability of the lens zonule can be difficult to determine using silt-lamp microscopy before surgery. This can lead to atypical clinical signs, resulting in lens subluxation being misdiagnosed as APAC (10). Zhang et al. found that among 2054 patients, 85 patients with lens subluxation were initially misdiagnosed as primary angle-closure glaucoma during their first visit to the hospital. Finally their diagnoses were modified to reflect lens subluxation (11). However, in clinics, common examinations such as ultrasound biomicroscopy (UBM) may not be well applied for all patients suffering from acute glaucoma attack as patients may not be able to cooperate with the examination due to eye pain, nausea, and vomiting or cannot undergo contact examinations due to corneal edema. Treatments such as using a miotic agent and laser peripheral iridotomy, which are commonly used in APAC, rarely control IOP to a safe level for patients suffering from ASAC-ZD. Prompt and appropriate treatment is critical to the prognosis. High IOP will lead to irreversible loss of vision and atrophy of the optic nerve if the cause of ASAC-ZD is not diagnosed immediately (12).

Therefore, there is a need for a quicker and more objective method of investigation to help clinicians in reducing the misdiagnosis rate of ASAC-ZD. In this study, we describe the ocular characteristics of ASAC-ZD and APAC patients measured using the Zeiss IOLMaster 700 with the advantages of non-contact and quick scanning. By analyzing the ocular characteristics, the IOLMaster 700 can detect incisively the difference in parameters between ASAC-ZD and APAC patients, which may aid in the differential diagnosis.

## 2 Materials and methods

### 2.1 Study design and patients

A retrospective study was conducted at the Zibo Central Hospital, in Shandong, China. This study was approved by the Human Research Ethics Committee of the Zibo Central Hospital and was performed with adherence to the tenets of the Declaration of Helsinki. Written informed consent was obtained from all the patients.

The medical records of patients with APAC diagnoses between September 2021 and December 2023 were reviewed. We excluded any patients who were diagnosed with lens subluxation at the initial visit. Phacoemulsification and intraocular lens implantation combined with goniosynechialysis were performed on patients in both groups to increase IOP. All patients underwent comprehensive eye examinations, including best-corrected visual acuity, IOP measured using Goldman tonometry, slit-lamp microscopy, IOLMaster 700 biometry, and funduscopy. Visual acuity measurements were converted to logarithms of the minimum angle of resolution (log MAR) equivalents. Ophthalmological parameters were measured using IOLMaster assessments of axial length (AL), anterior chamber depth (ACD), standard deviation of anterior chamber depth (ACDSD), lens thickness (LT), and standard deviation of lens thickness (LTSD). The ACD difference was calculated by the ACD in the affected eye minus the ACD in the contralateral eye. The above examinations were conducted by an experienced ophthalmologist. All procedures were performed by the same experienced surgeon.

### 2.2 Inclusion and exclusion criteria

Data were collected from 73 patients with unilateral APAC eyes during their first visit to Zibo Center Hospital from September 2021 and December 2023 consecutively. Of these, 34 were initially misdiagnosed with APAC and later re-diagnosed with lens subluxation during UBM or surgery. Acute primary angle closure (APAC) was diagnosed based on symptoms and signs including elevated IOP, shallower ACD, acute eye pain, blurred vision, nausea and vomiting, ciliary or mixed congestion, corneal edema, loss of pupillary light reflex, and varying degrees of bilateral cataract symptoms. The ASAC-ZD criteria used in this study are as follows: (1) decreased vision and sudden onset of eye pain; (2) elevated IOP; (3) silt-lamp microscopy showing shallow anterior chamber depth and dilated pupils, disappearance of light reflex, and corneal edema in the affected eye; and (4) lens subluxation and rupture of zonular ruptures confirmed at the subsequent surgery. Patients with any history of corneal disease or surgery, as well as major retinal surgery or treatment, such as panretinal photocoagulation, macular pathology, uveitis, other types of glaucoma (such as pigmentary glaucoma), retinitis pigmentosa, and Marfan syndrome, were excluded from the study.

### 2.3 Statistical analysis

All data were calculated using GraphPad Prism 9.0.0. Continuous variables such as age, axial length, and anterior chamber depth are presented as mean ± SD. Categorical variables such as sex were compared using the chi-square test or Fisher’s exact test. Continuous variables based on the distribution were analyzed using an independent-sample t-test or Mann–Whitney U-test. Logistic regression analysis was used to identify the correlation of the anterior chamber date associated with ASAC-ZD. For diagnostic assessment, the data of ACD, ACDSD, and LTSD in the affected eye and the ACD difference between the affected eye and contralateral eye were calculated from the area under the receiver operating characteristic (AUROC) curves and were used to delineate between the ASAC-ZD and APAC groups. A *p*-value of <0.05 was considered to be statistically significant.

## 3 Results

### 3.1 Basic features

In the study, there were 34 participants in the ASAC-ZD group and 39 participants in the APAC group with affected eyes. Generally, patients in the ASAC-ZD group were younger than those in the APAC group, with respective mean ages of 63.74 ± 6.947 and 69.92 ± 9.345 years (*p* = 0.004). The APAC group was predominantly female, significantly more than the ASAC-ZD group (*p* = 0.0144). Five patients (14.7%) in the lens dislocation group had a history of trauma to the eye or the arch of the eyebrow, which was significantly more than in the APAC group (*p* = 0.0185). Only one patient (3%) in the study group had bilateral lens displacement and was excluded from the research (Table 1).

**Table 1:** Demographic characteristics of the patients enrolled in the study.

Variables	APAC	ASAC-ZD	*p*-Value
Number of subjects	39	34	
Age (y)^a^	69.92 ± 9.345	63.74 ± 6.947	0.004**
Sex (Male:Female)^b^	9:30	18:16	0.0144*
Eye (Right:Left)^b^	20:19	14:20	0.4821
Trauma History^c^	0	5	0.0185*

^a^Mann–Whitney U-test; ^b^chi-square test; ^c^Fisher’s exact test; *value with statistical significance (*p < 0.05; **p < 0.01; ***p < 0.001; and ****p < 0.0001).

APAC, acute primary angle closure; ASAC-ZD, acute secondary angle closure associated with zonular dialysis.

### 3.2 Biometric data of affected eyes

The biometric data of affected eyes for the ASAC-ZD and APAC groups are summarized in Table 2. IOP, AL, ACD, ACD SD, and LT SD in the affected eye along with the AL and ACD in the contralateral eye were notably different between the two groups. When compared to the APAC group, the ACD difference between the affected and contralateral eyes was significantly lower in the ASAC-ZD group.

**Table 2:** Ocular biometric data from both affected eyes of the ASAC-ZD and APAC group.

	Variables	APAC	ASAC-ZD	*p*-Value
Affected eye	BCVA^a^	1.072 ± 0.5735	1.021 ± 0.508	0.989
	IOP (mmHg)^c^	34.69 ± 12.83	30.97 ± 14.85	0.2595
	AL (mm)^c^	22.39 ± 0.7852	23.23 ± 1.168	0.0008***
	ALSD (μm)^a^	10.18 ± 4.033	10.29 ± 4.174	0.8635
	ACD (mm)^a^	2.120 ± 0.2986	1.889 ± 0.5167	0.0002***
	ACDSD (μm)^a^	7.605 ± 5.425	9.941 ± 6.120	0.0044**
	LT (mm)^b^	5.172 ± 0.4266	5.056 ± 0.3412	0.2104
	LTSD (μm)^a^	28.00 ± 19.52	39.79 ± 22.74	0.0119*
ACD difference (mm)^a^		−0.1249 ± 0.2349	−0.7306 ± 0.5332	<0.0001****

^a^Mann–Whitney U-test; ^b^t-test; ^c^corrected t-test. *Value with statistical significance (*p < 0.05; **p < 0.01; ***p < 0.001; and ****p < 0.0001).

APAC, acute primary angle closure; ASAC-ZD, acute secondary angle closure associated with zonular dialysis. BCVA, best-corrected visual acuity; IOP, intraocular pressure; AL, axial length; D, diopter; ACD, anterior chamber depth; ACDSD, standard deviation of anterior chamber depth; LTSD, lens thickness standard deviation. ACD difference was calculated by ACD in the affected eye minus ACD in the contralateral eye.

No significant differences in best-corrected visual acuity (BCVA) and IOP between the two groups were found (*p* = 0.989 and *p* = 0.2595). The AL of bilateral eyes in the ASAC-ZD group was significantly longer than that of the APAC group (*p* = 0.0008 and *p* = 0.0012, respectively). Overall, 25 (82.4%) patients had AL > 23.5 mm in the ASAC-ZD group, while 5 (12.9%) patients had AL > 23.5 mm. Compared to the APAC group, ACD was shallower in the affected eyes (range: 1.12–3.56 mm; *p* = 0.0002) but deeper in the contralateral eyes of the ASAC-ZD group (range: 1.87–3.77 mm; *p* < 0.001). LD in the APAC group in both eyes was thicker than that in the ASAC-ZD group but did not show a significant difference (*p* = 0.2104 and *p* = 0.0722, respectively). The ACDSD and LTSD of the affected eyes in the ASAC-ZD group were significantly higher than those in the control group (*p* = 0.00044 and < 0.0119, respectively). However, the contralateral eyes no longer showed significant differences between the two groups. There was no significant difference in ALSD between the two groups. In addition, the ACD difference in the ASAC-ZD group was greater than that in the APAC group (*p* < 0.001) (Table 2).

### 3.3 Biometric data of affected eyes compared to intereye (intraindividual)

Intereye (intraindividual) anterior parameters between the affected eyes and their corresponding contralateral eyes of the ASAC-ZD and APAC groups were compared, as shown in Table 3. The BCVA of the affected eyes was worse than that of contralateral eyes (all *p* < 0.0001), and the IOP of the affected eyes was higher than that of contralateral eyes in both groups (all *p* < 0.0001). There was a weak but statistically significant difference in ACD between the affected eyes and contralateral eyes in the APAC group (*p* = 0.0494). However, the ACD of the affected eyes was significantly shallower than that of the contralateral eyes in the ASAC-ZD group (*p* < 0.0001). Moreover, the ACDSD and LT SD in the affected eye showed statistically significant differences between the contralateral eyes of the ASAC-ZD (*p* = 0.0402 and *p* = 0.0002). However, in the ASAC group, the differences in ACDSD and LTSD did not reach statistical significance (Table 3).

**Table 3:** Comparison of ocular biometric data between contralateral eyes and affected eyes in the APAC group and ASAC-ZD group.

	Variables	Affected eye	Contralateral eye	*p*-Value
APAC	BCVA^a^	1.072 ± 0.5735	0.4795 ± 0.5262	<0.0001****
	IOP (mmHg)^b^	34.69 ± 12.83	12.77 ± 2.786	<0.0001****
	AL (mm)^b^	22.39 ± 0.7852	22.40 ± 0.8152	0.9635
	ALSD (μm)^a^	10.18 ± 4.033	10.16 ± 3.301	0.9358
	ACD (mm)^a^	2.120 ± 0.2986	2.248 ± 0.3171	0.0494*
	ACDSD (μm)^a^	7.605 ± 5.425	7.026 ± 3.018	0.5600
	LT (mm)^a^	5.172 ± 0.4266	5.162 ± 0.3735	0.9609
	LTSD (μm)^a^	28.00 ± 19.52	22.24 ± 14.41	0.3551
ASAC-ZD	BCVA^a^	1.021 ± 0.508	0.3912 ± 0.2923	<0.0001****
	IOP (mmHg)^a^	30.97 ± 14.85	13.21 ± 3.764	<0.0001****
	AL (mm)^a^	23.23 ± 1.168	23.09 ± 0.9127	0.7078
	ALSD (μm)^b^	10.29 ± 4.174	9.500 ± 4.099	0.4315
	ACD (mm)^a^	1.889 ± 0.5167	2.649 ± 0.4581	<0.0001****
	ACDSD (μm)^a^	9.941 ± 6.120	8.118 ± 5.324	0.0402*
	LT (mm)^b^	5.056 ± 0.3412	5.003 ± 0.3250	0.5147
	LTSD (μm)^a^	39.79 ± 22.74	21.18 ± 15.96	0.0002***

^a^Mann–Whitney U-test; ^b^t-test. *Value with statistical significance (*p < 0.05; **p < 0.01; ***p < 0.001; and ****p < 0.0001).

APAC, acute primary angle closure; ASAC-ZD, acute secondary angle closure associated with zonular dialysis. BCVA, best-corrected visual acuity; IOP, intraocular pressure; AL, axial length; D, diopter; ACD, anterior chamber depth; ACDSD, standard deviation of anterior chamber depth; LTSD, lens thickness standard deviation.

### 3.4 Logistic regression analysis of lens subluxation

The results of the logistic regression analysis were analyzed to further identify the risk factors for lens subluxation. According to the univariate model, age, AL, ACD, LTSD, and ACD differences exhibited a notable correlation with lens subluxation (all *p* < 0.05). In addition, age, ACD, and ACD difference remained significant in the multivariate model (Table 4).

**Table 4:** Univariate and multivariate logistic regression analyses for risk factors of acute secondary angle closure associated with lens subluxation.

Variables	Univariate		Multivariate	
	OR	*p*-Value	OR (95% CI)	*p*-Value
AL (mm)	0.3871	0.0003***	1.019 (0.4035–2.735)	0.9626
ALSD (μm)	0.9933	0.9083		
ACD (mm)	4.421	0.0169*	18.21 (1.406–578.4)	0.0491*
ACDSD (μm)	0.9270	0.0820	1.011 (0.8805–1.156)	0.8691
LT (mm)	2.262	0.1899		
LTSD (μm)	0.9741	0.0197*	1.042 (0.9961–1.093)	0.0847
ACD difference (mm)	66.06	<0.0001****	0.0006850 (6.505e−006–0.01924)	0.0003***

Factors with *p* < 0.10 in the univariate model (AL, ACD, and ACD difference) were applied to the multivariate model. *Value with statistical significance (*p < 0.05; **p < 0.01; ***p < 0.001; and ****p < 0.0001).

APAC, acute primary angle closure; ASAC-ZD, acute secondary angle closure associated with lens subluxation; AL, axial length; ACD, anterior chamber depth; ACDSD, standard deviation of anterior chamber depth; LTSD, lens thickness standard deviation; ACD difference was calculated by ACD in the affected eye minus ACD in the contralateral eye.

### 3.5 The receiver operating characteristic (ROC) curve

**Figure 1:**
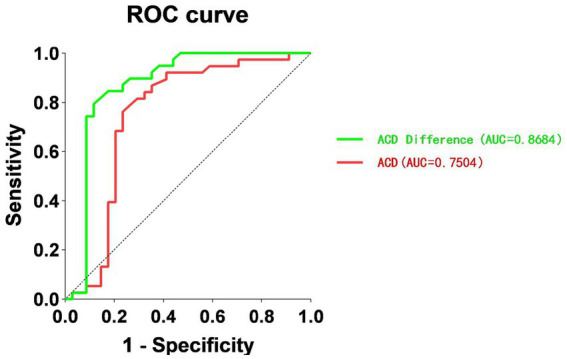
Receiver operating characteristic (ROC) curve for ACD and ACD difference, in relation to the diagnosis of ASAC-ZD. ROC, receiver operating characteristic; ACD, anterior chamber depth; ASAC-ZD, acute secondary angle closure associated with lens subluxation.

The receiver operating characteristic (ROC) curve was used to determine the potential diagnostic value of ACD difference and ACD between the lens subluxation and APAC groups, as shown in Figure 1.

The AUROC for ACD difference was 0.8684, and the ACD was 0.7504. The value of ACD at 1.93 mm was found to be the optimal cutoff point for the ASAC-ZD and APAC groups, with a sensitivity of 76.47% and a specificity of 76.32% (*p* < 0.0003). Moreover, the ACD difference at 0.225 mm had a sensitivity of 88.24% and a specificity of 79.49% (*p* < 0.001). The data are shown in Table 5.

**Table 5:** Cutoff value with sensitivity and specificity of ACD and ACD difference to be the optimal point for the ASAC-ZD and APAC groups.

Variables	AUC	Sensitivity	Specificity	Cutoff value	*p*-Value
ACD difference (mm)	0.8684	0.8824	0.7949	−0.2250	<0.0001
ACD (mm)	0.7504	0.7647	0.7632	1.930	0.0003

ACD, anterior chamber depth; ACD difference was calculated by ACD in the affected eye minus ACD in the contralateral eye.

## 4 Discussion

Lens subluxation is a multifactorial condition, with partial zonular ruptures identified as one of the main causes (7). When partial zonular ruptures, the lens shifts forward to attach to the iris, so that the lens and/or vitreous body can cause pupil block, leading to anterior chamber angle closure and increased IOP (8). However, some patients with lens subluxation do not have typical signs such as the absence of lens tremor or vitreous hernia (3). In our study, patients misdiagnosed with APAC did not exhibit typical signs of lens dislocation. Compared to the APAC group with denied trauma history, only five ASAC-ZD patients (14.7%) had a history of trauma to the eye or the arch of the eyebrow. The remaining ASAC-ZD patients denied any history of trauma, suggesting that the cause of the onset of lens subluxation may be more insidious and the diagnosis should be based on detailed clinical examination instead of solely medical history and description from patients.

However, administering a pupil dilation agent such as compound tropicamide, which should aid in diagnosis, may worsen high IOP in patients suffering from an unknown cause of acute glaucoma attack (13). In cases of combined corneal edema, making a differential diagnosis using slit-lamp examination can be challenging, necessitating the use of UBM. Zhang et al. identified that 4.1% of patients misdiagnosed with primary angle-closure glaucoma actually had lens subluxation, as determined by UBM (11). However, Chen et al. note that the operation of the UBM requires an experienced operator (3). Even an experienced operator cannot detect lens subluxation using UBM if the range of the suspensory ligament rupture is small. The misdiagnosis can lead to inappropriate treatment and be adverse to the management of high IOP (14). It has been reported that patients with ASAC-ZD have clinical characteristics such as a greater range of preoperative angle closure, longer operation time, and poor postoperative IOP control (11). Compared to lens extraction, which is widely used for APAC, the treatment of ASAC-ZD is more sophisticated, such as the implantation of a capsular tension ring, ciliary sulcus IOL placement, or sclera-fixated IOL depending on the extent of zonular weakness (15–17). The key points of treatment may be completely different from those of APAC.

To sum up, it is necessary to seek an objective and rapid examination method to distinguish APAC from ASAC-ZD to choose appropriate emergency treatments for acute glaucoma attack, which is the key to preventing blindness (18). The IOLMaster 700 with swept-source optical coherence tomography technology, which is widely used in clinics, has the advantages of non-contact and timeliness; it can measure biometric parameters such as ACD and LD, which play an important role in the diagnosis and analysis of lens subluxation (19). Using the IOLMaster, Jing et al. discovered that ACD < 1.4 mm can be highly indicative of lens zonular ruptures (7). By analysis of anterior segment parameters, Wang et al. reported that the angle between the iris and lens and iris non-anterior curvature are sensitive and characteristic indicators that indicate a clinical diagnosis of ASAC-ZD (18). Xing et al. discovered that the data from the anterior segment such as the relative position of the lens, anterior chamber depth, and aqueous depth can be indicators to distinguish APAC from ASAC-ZD (8). Therefore, we analyzed the comprehensive anterior segment parameters measured using the IOLMaster 700 to make a diagnosis between ASAC-ZD and APAC.

In our study, patients in the ASAC-ZD group presented acute glaucoma attack signs such as worse BCVA and higher IOP than the contralateral eyes, which were similar to the APAC group. The APAC group exhibited characteristics of being female-dominated, older age, shorter AL, and thicker lenses, consistent with previous studies. In contrast, the ASAC-ZD group was younger and showed no significant sex difference (20, 21). For anterior segment parameters, the ASAC-ZD group patients had significantly longer and normal ALs but a shallower ACD in the affected eyes compared to the APAC group in our study. In contrast, in the contralateral eyes of the ASAC-ZD group, ACD was deeper than the affected eyes in both groups along with longer AL. Chen et al. found that there was a positive correlation between longer AL and deeper ACD in China (22). This abnormal correlation between ACD and AL in our results indirectly validates that the lens position shifts forward in the ASAC-ZD group. This shift may lead to asymmetrical anterior chamber depth between both eyes in the ASAC-ZD group and a shallower ACD compared to the APAC group. According to previous studies, this asymmetry in ACD may be due to zonular ruptures, which can cause lens protrusion by increasing the curvature of the anterior lens capsule and changing the position of the lens. This results in the iris being pushed forward, creating a shallower ACD. The increased contact between the iris and lens causes disruption of aqueous humor circulation, leading to pupillary obstruction and inducing acute angle closure (15, 23). Apart from ACD, we found that the ACD difference in the ASAC-ZD group was larger than that in the APAC group. In a study of patients suffering from lens subluxation, the affected eye had a shallower anterior chamber angle compared to the contralateral eye, resulting in a relatively larger ACD difference. In contrast, the APAC group had narrow anterior angles in both eyes, and the ACD difference was instead smaller (5). The ACD difference of patients in our study showed a similar result as described in previous studies. Lin et al. found that the ASAC-ZD group consistently exhibited a clinical feature as asymmetrical ACD between the bilateral eyes (10). In our study, the ACD difference at 0.225 mm and ACD at 1.93 mm were found to be the cutoff values for lens subluxation. The ROC curve analysis indicated that ACD difference and ACD can serve as predictive markers with good value for lens subluxation patients. Jing et al. discovered that both ACD differences and ACD can be highly indicative of lens zonular ruptures, with a cutoff value of 0.63 and 1.4 mm using the IOLMaster (7). Chen et al. found a significant correlation between abnormally elevated ACD differences and lens subluxation through a combination of IOLMaster and UBM examination, with a cutoff value of 0.235 mm (3). Xing et al. suggested that when a patient has an acute attack of glaucoma, a monocular ACD of less than 1.25 mm is highly indicative of abnormality in the lens zonal hypertension or relaxation (8). According to the results, an asymmetric ACD resulting from a shallower ACD in one of the eyes combined with a normal AL in the unknown cause of APAC attack patients can be an important feature in the diagnosis of lens subluxation.

It was interesting to note that the ACDSD and LTSD of the affected eyes in the ASAC-ZD group were significantly higher than those of the APAC group and ASAC-ZD contralateral eyes. However, the ACDSD and LTSD in the contralateral eye showed no statistically significant difference between the two groups. The results can be explained by the mechanism of the IOLMaster 700 working as an ophthalmic biometer based on swept-source optical coherence tomography (24). It can scan from six different directions (0°, 30°, 60°, 90°, 120°, and 150°) to measure the parameters of the anterior segment, utilizing penetration, absorption, and reflection of light at various interfaces. The IOLMaster 700 can obtain six values such as ACD to automatically calculate the average value and standard deviation. Wang et al. discovered that lens subluxation patients presented an increased distance between the ciliary process and the crystal equator using UBM (9). In the event of zonule ruptures, the lens is shifted toward the quadrant with the normal zonules due to imbalanced forces, resulting in strong traction. As a result, the area of contact between the posterior surface of the iris and the anterior surface of the lens increases, the iris lens angle disappears, and the iris loses its normal convexity due to the extrusion by the dislocated lens (18, 25). Considering these factors, we suggested that the zonular ruptures enable the lens to not be in a centered position and a certain quadrant of the anterior chamber with different depths. By analyzing the crystal positions of patients misdiagnosed with APAC during their first visit, Xing et al. found that even if there is an occult lens subluxation, the rupture of the lens band has a significant impact on the position of the lens (8). When the IOL Master 700 collects anterior chamber data from six directions because of the change in lens position, each value will perform a larger difference, which will result in a higher standard deviation compared to the eyes containing a centered lens without rupture of the zonule in the contralateral eyes and the both eyes in APAC. Attention to the abnormal increase in ACDSD and LTSD in patients suffering from acute angle closure can provide more diagnostic evidence for lens subluxation. When an artificial slit-lamp examination cannot diagnose hidden lens dislocation, the IOLMaster can repeatedly measure LD and ACD in different directions, which can detect abnormal increases in ACDSD and LTSD and assist in diagnosis.

There are some limitations to our findings. First, the number of our patients was relatively small. Therefore, further confirmation through cumulative cases is needed. Second, a certain period of follow-up should be conducted to monitor postoperative intraocular pressure, changes in the number of corneal endothelial cells, anterior chamber depth, and atrophy of the optic nerve.

In summary, secondary glaucoma caused by lens subluxation is commonly misdiagnosed as APAC; as a result, symptoms and signs such as elevated intraocular pressure, corneal edema, and severe eye pain are not specific. Therefore, it is particularly recommended to analyze the clinical and ocular feature data of patients initially diagnosed with APAC. After evaluating the biometric parameters, our results indicate that an asymmetric ACD combined with a normal AL can be a potential predictive factor for identifying lens subluxation. Furthermore, abnormal increases in ACDSD and LTSD may aid in diagnosing ASAC-ZD. Our findings could help differentiate between APAC and ASAC-ZD, potentially reducing the probability of initial misdiagnosis and assisting in selecting the correct surgical methods and medications.

## Data Availability

The raw data supporting the conclusions of this article will be made available by the authors, without undue reservation.
